# 

**Published:** 1868-05

**Authors:** 


### THE AMERICAN JOURNAL OF DENTAL SCIENCE,

EDITED BY

A. SNOWDEN PIGGOT, M. D.,

AND

F. J. S. GORGAS, M. D., D. D. S.

VOL. 2.—THIRD SERIES.

Surgeon Genl's Office LIBRARY. 39612 Washington, D.C.

BALTIMORE:

SNOWDEN & COWMAN.

LONDON:

TRUBNER & CO., 12 PATERNOSTER ROW.

1867.

W
1
AM 45 M

3375 c

### INDEX TO VOL. II.---THIRD SERIES.

Absinthe Drinking in Paris, - - - - - - 245
Absinthism, - - - - - - - - - - - - 40
Academy of Dental Science. Annual Meeting of - 329
A Degree Easily Obtained, - - - - - - - 49
A Desirable Invention, - - - - - - - 311
A Good Cement, - - - - - - - - - 405
A Monster Tumor, - - - - - - - - 95
A New Rubber Company, - - - - - - 335
A Novel Invention, - - - - - - - - 503
A Self Acting Blow Pipe, By Wm. S. Russell, D.D.S. - 473
A Wonderful Skull, - - - - - - - - 193
Address before Tenn. State Dental Association
By W. H. Morgan, D.D.S.,M.D., - - - 506
Administration of Nitrous Oxide Gas.
By G. W. Massamore, D.D.S., - - - - - 328
Adulteration of Food, - - - - - - - - 456
Affections of the Nervous System, Dependent on Diseases
of Teeth. - - - - - - 304. 336
Alopecia. Causes of - - - - - - - 96
Aluminium Solder, - - - - - - - - 359
Alveolar Abscess, - - - - - - - - 376
Amaurosis from Neuralgia of Dental Nerves, - - 497
Amalgam Fillings. A Method for Removing, - - - 104
American Dental Association, - - - - - - 102
American Dental Association. Proceedings of Eighth
Annual Meeting of - - - - - - 266
Ammonia Iron Alum, - - - - - - - - 398
Anæsthesia, and Mode of Action of Anæsthetics, - 406
Anæsthetic. A New - - - - - - - - 154
Andrews, Prof. E. - - - - - - - - 440
Aneurismal Varix, By Jno. N. Monmonier, M.D. - - 231
Animal Quinoidin. The Doctrine of - - - - - 448
Answers to Querists, - - - - - - - - - 23
Treatment of Teeth Rendered Sensitive by Mechanical
Abrasion, - - - - - - - - 23
Extracting Teeth for Pregnant Females, - - 24
Antidote for Strychnia. - - - - - - - 38
Apparent Exercise of Volition During Anæsthesia, - 497
Arnica. External Use of - - - - - - - 99
Artificial Digestion, - - - - - - - - 356
Artificial Palates. A New Method of Constructing
By Dr. Wm. Suersen, Sen. - - - 1

Artificial Respiration in Cases of Opium Poisoning, - 500
Association of the Colleges of Dentistry, By J. Taft, D.D.S. 49
Atmospheric Plates. A New Method for Constructing
By N. T. Folsom - - - - - - - 179
Baltimore College of Dental Surgery, - - - - - 358
Baltimore College of Dental Surgery. Changes in the Faculty
of the - - - - - - - - - 553
Baltimore College of Dental Surgery. Commencement week
at the - - - - - - - - - 581
Baltimore College of Dental Surgery. Annual Circular of 599
Beers, Dr. W. George - - - - - - - - 434
Bibliographical Notices, 48. 99. 156. 207. 258. 309.407.455.501.598
Plastic Surgery, - - - - - - - - 48
Taft's Operative Dentistry, - - - - - 99
Canada Journal of Dental Science, - - - 156
Revista Medico–Quirurgica Y Dentistica DeLos Sres, 207
Loomi's Lessons in Physical Diagnosis, - - 258
White's Dental Materia Medica, - - - 309
Lindsay & Blackiston's Physicians' Visiting List for
1869, - - - - - - - - - 309
Lea's Catalogue of Med. and Surg. Publications.
Illustrated, - - - - - - 310
Richardson's Mechanical Dentistry, - - 407
Baltimore Medical Bulletin, - - - - - 407
Medical Gazette, - - - - - - - 407
Mackenzie on the Laryngoscope, Loss of Voice, &c. 501
Cleaveland's Pronouncing Medical Lexicon, - 501
Wythe's Pocket Dose Book, - - - - 502
Shaw's Treatise on Odontalgia, - - - 502
Missouri Dental Journal, - - - - - - 455
Robertson on Extracting Teeth, - - - 455
Hance's Physicians Med. Compend & Formula - 455
Chemical News, - - - - - - - 456
Heath on Injuries and Diseases of the Jaws, - 598
Bleaching a Necrosed Tooth, - - - - - 377
Browne, Prof. Rufus King, M.D., - - - - - 183
Cancer of Antrum, - - - - - - - 450
Cancrum Oris. Diagnosis of - - - - - - 406
Carbolic Acid in Burns, - - - - - - 401
Caries of the Teeth. The Cause of - - - - 154
Case of Death from Chloroform, By E. A. Clark, M.D., 446
Cases from the Note Book of A. Preterre, - - - 132
Causation of Phthisis, - - - - - - - 40
Chaffee, Dr. W. N. - - - - - - - - 532
Character and Uses of Saliva. Correction,
By H. S. Chase, D.D.S.,M.D. - - - - - 334
Chase, H. S., D.D.S. - - - - - - - 334
Chlorine Gas in Toughening and Refining Gold, - - 548
Chloroform to Cure Sneezing - - - - - - 206

Clark, E. A. M.D., - - - - - - - - 446
Cohnheim's and Virchow's Views on the Origin of Pus Corpuscles. By R. K. Brown, M.D., - - - - 183
Commencement Week at Balto. College of Dental Surgery. 581
Compound Fracture of the Lower Jaw, - - - 254
Colorless Tincture of Iodine, By Hubert Primm, Ph. D., - 93
Corn Meal, - - - - - - - - 504
Cosbey, Benjamin Franklin, D.D..S, - - - - - 66
Carious Exostotic Growth, - - - - - - 405
Crecchio, Prof. M. - - - - - - - - - 391
Creasote. Characteristics of the Different Varieties of - 37
Crook, Wm. M.D., - - - - - - - 348
Cutler, Andrew S. M.D., - - - - - 181. 220
Cutler, S. P. D.D.S., M.D., - - - - 261. 324. 362
Cystic Tumor of the Lower Jaw, - - - - - 250
Death from Carbolic Acid, - - - - - - 355
Death from Chloroform, - - - - - - 155
Death from Cold. On, By Prof. Crecchio, - - - 391
Death from Extraction of a Tooth. By C. W. Westmoreland, D.D.S., - - - - - - - 475
Death from Hæmorrhage Following Extraction of a Tooth. By Mr. O. Salomon, - - - - 521
Dental Colleges, - - - - - - - 310. 553
Dental College Commencements, - - 22. 581. 586. 587
Dental Education. By R. B. Winder, D.D.S., - - - 575
Dental Pulp. Treatment of - - - - - - 103
Dental Tumors, - - : - - - - - 367
Dental Tumors, ByAM. Forget, - - - - 223
Dentition. Early - - - - - - - - 597
Dentistry as a Fine Art. By Prof. N. W. Kingsley, - 161
Dickson, John, M.D., - - - - - - - 144
Digestion, By John W. Holt, D.D.S. - - - 410. 465
Digitalis, - - - - - - - - - - 41
Dissections, - - - - - - - - - 453
Division and Excision of Nerves. - - - - 403
Divisibility of Matter, - - - - - - - 200
Dr. Morgan's Address, - - - - - - 553
Dunn, Prof. Chas. E. - - - - - - - 52
Eames, W. H., D.D.S. - - - : - - 482
Early Trials of Experimental Physiologists, - - - 204
Effects of Alcoholic Stimulants upon Action of Anæsthetic Agents, - - - - - - - - - 156
Effects of Darkness and Silence, - - - - 203
Electrical Probe, - - - - - - - - - 251
Employment of Glycerine Tannin, - - - - 594
Encouraging, - - - - - - - - - 456
Epilepsy. Extraordinary Cure of - - - - 597
Epulis or Fibrous Tumor of the Gums. - - - - 378
Evans, J. D'Oyley, D.D.S. - - - - 84. 140. 232
Evans, Thos. W, M.D., D.D.S., - - - - - - 525

Examinations of Blood Stains, - - - - - - 29
Excision of Breast, under N. O. Gas, - - - - - 38
Excision of Upper Jaw, - - - - - - - 437
Exploration of the Mouth. By W. H. Wait, D.D.S., - 192
Extensive Removal of Bones of the Face, - - - 355
Extirpation of the Right Parotid Gland. By Wm. Crook, M.D. 348
Extracting Teeth for Pregnant Females, - - - 24
Extraction of Difficult Teeth, - - - - - - - 414
Fang Operations. Diagnosis in, By Benj. F. Cosby, D.D.S., 66
Fatal Hæmorrhage After Extraction of a Tooth, - - 155
Field, S. M., D.D.S., - - - - - - - - 136
Filing Teeth, By James Trueman, D.D.S. - - - 476
Filling Frail Cavities. By Dr. W. George Beers, - - 434
Filling Pulp Cavities, - - - - - - - 103
Folsom, N. T. - - - - - - - - 179
Foreign Bodies in the Ear. By H. L. Shaw, M.D., - 86
Foreign Bodies in the Æsophagus, - - - - 197
Forget. AM., - - - - - - - - 223
Form of Delegate's Certificate to Am. Dent. Association, 422
Fracture of Nasal Bones, &c., - - - - - - 496
Fracture of the Lower Jaw. Treatment of
By Thomas B. Gunning, D.D.S., - 53. 106. 172. 214
Fracture of Upper and Lower Jaws. A Case of
By T. O. Oliver, - - - - - - - 68
Functions of the Pancreas, - - - - - - - 388
Fungous Growth of Nerve, By C. W. Westmoreland, D.D.S. 474
Gamgee, S. F.R.C.S., - - - - - - - - 539
Goodall's Patent Elastic or Spring Plate, - - - 469
Goodyear Rubber Cases, - - - - - - 160, 422
Gums. Pathology of - - - - - - - - 190
Gunning. T. B. M.D.,D.D.S., - - 10. 53. 106. 172. 212. 214
Heath. Christopher F.R.C.S., - - - - - - 393
Hæmorrhage after Extraction of Teeth, - - - 408
Hæmorrhage after Extraction of a Tooth - - - 155
Hare Lip. Hereditary Nature of - - - - - - 257
Holt. John W., D.D.S. - - - - 410. 465
Horsford. Prof. E. N., - - - - - - - 488
How to Extract Silver from Lead, - - - - - 36
How to Utilize Leeches, - - - - - - - 354
Howard, Prof. E. Lloyd, M.D., - - - - - 554
Howard, R.P., M.D., - - - - - - - 32
Hudson, R. N., D. D. S., - - - - - - 69
Hypertrophy of the Right Half of the Body of a Child, 400
Hypodermic Use of Morphia during Anæsthesia, - - 207
Hypophosphites in the Tooth-ache of Pregnancy, - 500
Illinois State Dental Society, - - - - - - 76
Infantile Convulsions, By John Dickson, M.D., - - 144

Influence Exerted by Anæsthetics on Brain and Nervous System, - - - - - - - - - - 44
Influence of Electricity on Muscular Fibre of Vegetable Life, and on Nutrition, - - - - - - 46
Influence of Weather upon Sickness, - - - 249
Injurious Effects of Sewing Machines Prevented, - - 404
Insalubrity of Cast Iron Stoves, - - - - 256
Insecticides, - - - - - - - - - 356
Insertion of an Artificial Crown on Roots of a Superior Bicuspid Tooth, - - - - - - 418
Intermittent Pulse and Palpitation, - - - - 258
Iodoform, - - - - - - - - - - 255
Irregularity, - - - - - - - - - 375
Keratitis, - - - - - - - - - - 98
Keesee, Geo. F., D.D.S. - - - - - - 565
Kingsley. Prof. Norman W. - - - - - 161
Lancing the Gums in Children, - - - - - 206
Large Salivary Calculus, - - - - - - 498
Leeches. How to Utilize - - - - - - 354
Letters from Paris. By J. D'Oyley Evans, D.D.S. 84.140.232
Letter from Josiah Bacon, Esq., Treas. Goodyear Rubber Company, - - - - - - - - - 235
Letter from Thos. W. Evans, M.D.,D.D.S, Paris, - - 525
Letter from W. O. Kulp, D.D.S. - - - - - - 585
Liquefying Laughing Gas, - - - - - - 402
Liquors. Adulteration of - - - - - - - 590
Local Anæsthesia. New Method of - - - - 47
Local Anæsthetics for Traumatic Tetanus, - - - 153
Loss of a Portion of Lower Jaw and Tongue. Artificial Replacement and Restoration of Articulation. By Geo. H. Perrine, D.D.S., - - - 345
Man's Antiquity in America, - - - - - - 454
Massamore, G. W., D.D.S., - - - - - - 328
Maury's Tooth Brushes, - - - - - - - 504
Maryland State Dental Society. By S. M. Field, D.D.S. 234. 379
Medication. Hypodermic, By Prof. Edward Warren, M.D. 588
Medullary Cancer of the Jaw of a Child. By Christopher Heath, F.R.C.S., - - - - - - 393
Menecier, Dr. Chas. - - - - - - - - 458
Microscopy of the Dental Tissues. By S. P. Cutler, M.D., D.D.S., - - - - - - 261. 324. 362. 514
Mode of Dividing Glass, - - - - - - - 595
Monmonier, John N., M D., - - - - - 134. 231
Morgan, W. H., M.D., D.D.S., - - - - - - 506
Movement. Origin and Nature of - - - - 43
Mucus. By Andrew S. Cutler, D.D.S., - - - - 181
Nasal Therapeutics, - - - - - - - - 452

Necrosis of Inferior Maxillary. By Wm. Mason Turner, M.D. 486
Nerves of the Neurillemma, - - - - - - - 353
Nervous Influence on Salivary Secretion, - - - - 385
Neuralgia. By Professor Trousseau, - - - - - 189
New Method of Constructin g Rubber Plates.
By W. H. Evans, D.D.S., - - - - - 482
New Method of Local Anæsthesia, - - - - - - 47
New Modes of Preparing Objects for the Microscope, - 493
New Styptic, - - - - - - - - - - 207
Nitrous Oxide Gas. By R. N. Hudson, D.D.S., - 69
Nitrous Oxide, - - - - - - - - - - 151
Nitrous Oxide Gas as an Anæsthetic. By H. - - 333
Nitrous Oxide Gas. By Geo. W. Massamore, D.D.S., - 328
Nitrous Oxide Gas, - - - - - - - - - 259
Nitrous Oxide Gas. By Thos. W. Evans, M.D.,D.D.S., . 525
Nitrous Oxide as an Anæsthetic, - - - - - 494
Noel, Prof. Henry R. - - - - - - - - - 314
Notes from Dental Practice - - - 375, 414, 473. 571
Filling Teeth, - - - - - - - - 571
Irregularity, - - - - - - - - - 375
Alveolar Abscess, - - - - - - - - 376
Alveolar Abscess with Facial Fistulæ, - - 573
Bleaching a Necrosed Tooth, - - - - - 377
Epulis or Fibrous Tumor of the Gums, - - 378
Extraction of Difficult Teeth, - - - - - 414
Insertion of Artificial Crown on Roots of a Superior
Bicuspid Tooth, - - - - - - 418
A Self Acting Blow Pipe. By W. T. Russell, D.D.S. 473
Fungous Growth of Nerve. W. W. Westmoreland,
D.D.S., - - - - - - - - - 474
Death from Extraction of a Tooth. By W. W.
Westmoreland, D.D.S., - - - - - 475
Size of Roots of Teeth. Methods for Determining
By O. Salomon. - - - - - - - 574
Notes of Clinical Practice, - - - - - - 486
Obituary Notices. Isaac McKim McCurley, D.D.S. - 505
Dr. A. W. Allen, - - - - - 505
Professor A. Snowden Piggot, M. D. 549
Odontomes, - - - - - - - - - - 205
Ointment. Treatment of Fracture by - - - - - 596
Oliver, T. O. - - - - - - - - - - 68
Oxy-Chloride of Zinc over Exposed Nerves. - - 50
Oxygen Mixture. A New Anæsthetic Combination.
By Prof. E. Andrews, - - - - - - - 440
Ozone, - - - - - - - - - - - - 40
Parasites of the Cheek, - - - - - - - - 401
Pennsylvania College of Dental Surgery, - - - - 586

Peripheral Termination of the Motor Nerves, - - - 42

Permanent Union of the Maxillary Bones.

By Prof. Edward Warren, M. D., - - - 519

Perrine, G. H., D.D.S., - - - 345

Philadelphia Dental College, - 587

Physiological Action of Muscles of Lower Jaw.

By Thos. B. Gunning, D.D.S. - - 10

Plastic Gold. By John G. Mackintosh, D.D.S., - - 127

Plastic Gold. An Explanation, - - 360

Plastic Surgery, - - 48

Poisons Found in Snuff, - - - - - - 202

Poisoning by Carbolic Acid, - - - 152

Premature Interment, - - 257

Preservation of Vaccine Virus in Glycerine, - 204

Primm. Hubert Ph. D. - - - - - 93

Proceedings of Association of the Colleges of Dentistry.

By J. Taft, D.D.S. - - - - - 71

Proceedings of American Dental Association, - 266

Protagon - - - - - - - - 130

Protest of Alumni of Penn. College of Dental Surgery, 143

Protoxide of Nitrogen as an Anæsthetic, 494

Publishers' Notice, - - - - - - - 312

Recitations in Medical Colleges, - 408

Refining Gold, - - - - - 500

Removal of the Entire Tongue. By S. Gamgee, F.R.S. - 539

Removal of a Portion of Superior Maxilla for a Fibro.

Cartilaginous Tumor, - - 498

Resection of the Head of Humerus.

By Jno. N. Monmonier, M.D. - 134

Replacement of a Tooth, - - - 157

Rose Pearl Base, - - - - - 600

Ross, Harrison, D.D.S. - - 190

Rubber Suit. Another - - - - 359

Russell, Wm. T., D.D.S. - - - 474

Salomon, Mr. O. - - - - - 574

Salter, Mr. - - - - - - - 304, 336

Sea Sickness, - - - - - - 505

Second Sight, - - - - - - 598

Shaw, Henry L., M.D. - - - 86

Simple Cavities. Treatment of By W. W. Waite, D.D.S. 531

Simple Synthesis of Urea, - - - - 253

Salomon, Mr. O. - - - - - - 521

Singular Cause of Death, - - - - - 206

Smith, Stephen, M.D. - - - - - - 25

Source of Free Hydrochloric Acid in the Gastric Juice.

By Prof. E. N. Horsford, - - - 488

Southern Dental Association - - - - 358. 502

Sponge Tents Carbolized, - - - - - - - - 201
Spray of Ether for Relieving Pain, - - - - - 201
Staphyloraphy, - - - - - - - - - - 202
Staphyloraphy. By W. Mason Turner, M.D. - - 486
Staphyloraphy in Case of Congenital Fissure of Hard
and Soft Palates. By R. P. Howard, M. D. - 32
State Laws to Regulate the Practice of Dentistry, - - 157
Steel, - - - - - - - - - - - 596
Stein. A. W., M.D., - - - - - - - 231
Structure of Tactile Corpuscles Demonstrated, - - 497
Structure and Functions of the Lymphatics - - 120
Stuck's Rubber Plates, - - - - - - - - 600
Students Address. By George F. Keesee, D.D.S. - - 565
Styptic Paper, - - - - - - - - - 402
Suersen. Dr. Wilhelm, Sen., - - - - - - 1
Sulphuric Acid in Living Animals, - - - - - 39
Surgery of the Hand. By Stephen Smith, M.D., - - 25
Syphilis Communicated by a Kiss, - - - - - 236
Taft. Prof. J. - - - - - - - - - 71
Taking Impressions for Partial Sets of Teeth.
By Dr. W. N. Chaffee, - - - - - - 532
Tartar. By Andrew S. Cutler, D.D.S., - - - - 220
The Cellular Theory. By Dr. A. W. Stein, - - - 239
The Defamatory Article in the Dental Times, - - 208
The Foot, - - - - - - - - - - 254
Tennessee State Dental Association. By W. S.
Arrington, D.D.S., - - - - - 302, 450
The Close of II. Volume, - - - - - - - 599
The New Anæsthetic (?) Nitrous Oxide, - - - - 151
The Niagara Meeting, - - - - - - - - 310
The Printing of the Journal, - - - - - - 159
The Rubber Case at Canandaigua, N. Y., - - - 160
The Rubber Patents, - - - - - - - 409
The Rubber Question, - - - - - - - - 105
The Stains of Iodine, - - - - - - - 598
The Use of Veratrine in Neuralgia, - - - - - 206
Thymic Acid, - - - - - - - - - 457
Time of Meeting of American Dental Asssociation. - 104. 160
To Test Purity of Water, - - - - - - - 357
To Make Linen Fire Proof. - - - - - - 28
Trousseau, Prof. - - - - - - - - - 189
Truman, James, D.D.S. - - - - - - - - 476
Tumors of the Maxilla and Amputation of this Organ.
By Dr. Chas. Menecier. - - - - - - 458
Tumors of the Tongue and Pharynx. - - - - 41. 252
Turner, Wm. Mason, M.D. - - - - - - - 486

Use of the Muscles of the Uvula in Deglutition.
By Thos. B. Guning, D.D.S., M,D. - - - - 212
Ventilation and Contagion, - - - - - - - 534
Valedictory Address. By Prof. E. Lloyd Howard, M.D. 554
Wait, W. H., D.D.S. - - - - - - - 192. 531
Warren, Prof. Edward, M.D. - - - - - 519. 588
Washington University of Medicine, - - - - 575
Westmoreland, C. W., D.D.S. - - - - - - 474. 475
What is Mucus? What is Pus? By Pof. H. R. Noel, M.D. 314
Winder, R. B., D.D.S - - - - - - - - 575
"With Brains Sir," - - - - - - - 98

### CORRESPONDENCE.

*Dental College Commencements. (Continued.)*

*New York College of Dentistry.*

The second annual commencement of this College was held on the 20th of March last in the Cooper Institute. A large audience was present and Dodworths' orchestral band enlivened the intermission. Prof. Kingsley the Dean of the Faculty read the annual report, and the degree of Doctor of Dental Surgery was conferred upon the following gentlemen, eight in number, by Prof. E. Parmly, the President of the College :

New York—Henry F. Blakeny, William Carr, Henry Dodin, Francis M. Odell, Sanford C. Barnum ; England—W. P. Ballard ; Michigan—James Parsons ; Massachusetts—J. Albert Kimbal ; South Carolina—W. C. Wardlaw, M.D.

Professors Parmly and Dunning made some appropriate remarks, and were followed by R. J. Hitchcock, D.D. who spoke of the great public benefits to be derived from dentistry, and from the developement and progress of this branch of the surgical science and art, and proved the beneficial influence of its advancement on the well-being of the human race.

The number of students in attendance upon the lectures of the course was forty-one.

*Philadelphia Dental College.*

The fifth annual commencement of this College was held on the 13th of March at Concert Hall. The degree of Doctor of Dental Surgery was conferred by Dr. R. Shelton Mackenzie, Secretary of the Board of Trustees upon the following gentlemen, twenty in number :

David W. Averill, Wisconsin ; Juan Garcia Bastida, Cuba ; Carlos Baron, Cuba ; John Henry Carter, Jr., England ; Diedrich Albert Claus, Germany ; Richard C. Dawkins, Kentucky ; Edward Duval, Cuba ; J. Lehman Eisenbrey, Pennsylvania ; Charles T. Frink, Massachusetts ; Edward L. Hewitt, New Jersey ; Richard Ker, Pennsylvania ; D. A. L. Laverty, Pennsylvania ; James F. Love, England ; William L. Maxton, Pennsylvania ; Jacob B. Morgan, Iowa ; George W. Obey, Ohio ; Joel E. Slegel, Pennsylvania ; Albion P. Stevens, New Hampshire ; S. Porter Shaw, Pennsylvania ; Robert R. Underwood, Pennsylvania.

The number of students in attendance upon the lectures of the course was forty-four.

---

*Answers to Querists.*

### TO READERS AND CORRESPONDENTS.

Communications intended for publication, and exchange journals and papers, should be addressed to the EDITORS OF THE JOURNAL, Baltimore, Md. All letters relating to the business of the Journal, or containing cash remittances, to be directed to Messrs. SNOWDEN & COWMAN. Articles for publication should be received by the editors at least three weeks previous to the first day of the month on which the number in which it is designed for them to appear, is issued.

### CONTENTS:

### ORIGINAL COMMUNICATIONS.

PAGE.

ARTICLE I.—Artificial Palates. By Dr. Wilhelm Suersen, Sen.................. 1

ARTICLE II.—On the Physiological Action of the Muscles which control and influence the Lower Jaw. By Thomas Brian Gunning, M.D.,D.D.S.............. 10

### CORRESPONDENCE.

Dental College Commencements.............................................. 22

Answers to Querists.—Treatment of Teeth rendered sensitive by Mechanical Abrasion.............................................. 23

Extracting Teeth for Pregnant Females.............................................. 24

### SELECTED ARTICLES.

ARTICLE III.—On the Surgery of the Hand. By Stephen Smith, M.D.............. 25

ARTICLE IV.—Examination of Blood-Stains.............................................. 29

ARTICLE V.—Staphylorrhapy in the case of Congenital Fissure of the Hard and Soft Palate. By R. P. Howard, M.D., L.R.C.S.E., etc.............................. 32

### MONTHLY SUMMARY.

How to Extract Silver from Lead.............................................. 36

On the Characteristics of the Different Varieties of Creosote.................. 37

To Make Linen Fire-Proof.............................................. 38

Antidote for Strychnia.............................................. 38

Excision of Breast; Anæsthesia by Nitrous Oxide Gas.............................. 38

Sulphuric Acid in Living Amimals.............................................. 39

Ozone.............................................. 40

Absinthism.............................................. 40

Causation of Phthisis.............................................. 40

Digitalis.............................................. 41

Tumors of the Tongue and Pharynx.............................................. 41

Peripheral Termination of the Motor Nerves.............................................. 42

Origin and Nature of Movement.............................................. 43

Influence exerted by Anæsthetics on the Brain and Nervous System.............. 44

Influence of Electricity on Muscular Fibre of Vegetable Life and on Nutrition.. 46

New Method of Local Anæsthesia, Applicable to the Extraction of teeth and other Operations in the Cavity of the Mouth.............................................. 47

### BIBLIOGRAPHICAL NOTICES.

Plastic Surgery.............................................. 48

### EDITORIAL DEPARTMENT.

The Association of the Colleges of Dentistry.............................................. 49

A Degree Easily Obtained.............................................. 49

Oxy-Chloride of Zinc over Exposed Nerves.............................................. 50

The Action of the Philadelphia Dental College.............................................. 51

Prof. Charles E. Dunn.............................................. 52

☞ The American Journal of Dental Science will contain fifty pages of reading matter in each number, making a volume of not less than *Six Hundred* pages;

**EXCLUSIVE OF ADVERTISEMENTS.**

